# Soldering

**Published:** 1896-08

**Authors:** H. J. Goslee

**Affiliations:** Chicago, Ill.


ARTICLE V.

### SOLDERING.

DR. H. J. GOSLEE, CHICAGO, ILL.

The essential elements of soldering are: The thorough removal of all oxidation from the surfaces to be united; contact or apposition of the parts; the use of a flux on the surfaces of both metal and solder, which permits the latter to flow readily, and of union between them, by preventing oxidation; and the heating up of the object to be soldered—whether it be in an invesiment or not—to or near the degree of heat required to fuse the solder, before any attempt is made to do so.

Now, the definition of solder, as we understand it, is a combination of metals which fuse lower than the highest fusing component part; zinc being mostly used in the ordinary gold solders as the baser incorporate, by which the fusing point of the mass is reduced and regulated, imparting also increased properties of flowing. Consequently, a 20-k. solder, for instance, will fuse lower than plate of the same karat, while it may, of course, be done with perfect ease and to good advantage when two or three solderings are required, as by lessening the possibility of unsoldering parts already attached, during the subjection to subsequent soldering. This enables you to accomplish a desired result by using as high a karat as possible in finishing.

Perhaps paramount among the difficulties encountered in, or connected with, the soldering of plate or crown-and bridge-work, it is apparent unavoidable fracturing of porcelain facings; and while it now seems probable that the coming work of this kind may, perhaps, be done without subjecting the porcelain to the heat of soldering, "but by the method already in vogue, apparently to some considerable extent, of simply soldering the backings which had previously been perfectly fitted and adapted, in their respective or relative positions, and subsequently retaining the facings by means of cementing and riveting;" it is, at the same time, perhaps, just as well to consider for a moment the cause of the frequent checking of facings, for there seems to be no imperative reason why it cannot often be avoided, though we are aware that some of our best men do not hesitate to make broad assertion "that few facings are ever entirely devoid of checks after soldering," and contend that if they are not noticeable at the time, they may be conspicuously so afterward.

It must be remembered that a porcelain facing in itself presents two substances—the porcelain or feldspar, and the platinum pins—each of which is affected entirely differently by the heat necessary to solder, the former absorbing and retaining the heat for a considerable time, and the latter, while absorbing it readily, gives it off or cools with equal rapidity. Consequently, when a tooth properly backed, is to be soldered, too much care cannot be exercised in cautiously and evenly distributing the heat from the beginning of its application, so that the porcelain which is usually farthest away and covered or protected by an investment material, receives it in equal proportion simultaneously with the metal; otherwise the metal receiving the bulk of the heat first rapidly, through the medium of the platinum pins, conducts it to the porcelain before the latter is correspondingly and sufficiently heated to avoid uneven expansion.

Acknowledging this, then, to be one, and perhaps the main cause, it is evident that it can be avoided by so regulating the application of the heat that the investment, which is usually a poor conductor, receives the most of it, or entirely so, till the whole shall have reached a high degree; which, having come from the outside, as it were, must have been equally and evenly distributed; and furthermore, as a precautionary means, the platinum pins should always be cut off as close as possible to the backing, leaving enough remaining to split or rivit; for if left standing out straight or bent over on the backing, they must necessarily receive most of the heat from the blow-pipe.

Great care is also necessary in the preparation of a facing to receive its backing, and in the perfect adaptation of the backing, that no overhanging edges of the metal be left on the porcelain, as this will invariably result in a fracture of the numerous small checks along the edges of the facing so neglected, and due to the impingement on the porcelain. And of equal importance is the arrangement of the teeth in alignment, that sufficient space be left between them to allow physical change in the metal on cooling. After the soldering is completed, the utmost care should be exercised in excluding any draught of air, which may have a tendency to prevent the contraction of porcelain and the metal taking place gradually. No attempt should be made to remove the case from the investment till this may have taken place and both are cool.

The extravagant and injudicious use of borax is another cause of numerous cracked facings, as it fuses lower than solder, and, owing to the expansion of the average investment material, it runs over the edges of the backing on the surfaces of the porcelain, and results in fracturing by giving off its heat and contracting much sooner.

The use of perhaps the most commonly-used investment material, consisting of pumice stone and plaster of Paris, presents very objectionable features. It is a poor conductor of heat, expands very readily, and, unless pro. tected by a ferrule of metal, or by wiring tightly together, or by being much larger in bulk than either desirous or necessary, it invariably cracks open or falls apart, rendering liable the exposure of the heated facing to the air, which results in fracturing. The pumice stone, like borax fuses lower than the ordinary solders, and, if in contact, adheres to the surface of the teeth, and cools and contracts much sooner.

When checks or cracks afterward occur it is generally due, not to the fracture having been there since the tooth was subjected to the soldering, but more likely to negligent or poor adaptation of its backing, rendering in itself no protection whatever to the facing from the continued strain brought to bear on it in the force of mastication. I think it is safe to say that there are not one-fourth of the facings used, whether they be on plates or crowns or bridges, that are properly protected to withstand the ravages of this powerful force.

To see a facing badly checked or entirely broken off of a crown or bridge after it has been worn for some little time is by no means an uncommon occurrence with most of us, but to attribute this to the soldering seems hardly consistent, when, as a usual thing, all we have to do is to examine the broken facing to see that the facing has had little or no protection along the cutting edge. When we consider the force to which a facing is subjected in masticating, and remember it is retained by means of its attachment in a position both rigid and unyielding to the slightest extent, how can we overlook so important an essential as providing for the proper protection of them against strain? This can be accomplished with very little more work and much more gratifying results.

Crown-and bridge-work would prove less laborious and more satisfactory to both dentist and patient, if this evil were avoided.

To accomplish this the facing should first be ground to assume its proper position and fit accurately, after which the lingual portion of the cutting edge should be so ground as to produce a sharp but smooth angle along the edge, the bevel extending to about one-half the distance to the pins; then the lateral sides should be slightly ground to give a smooth marginal edge to which the backing may be finished nicely and without danger of overhanging edges.

Use preferably pure gold from twenty-nine to thirty gage, and, after burnishing to fit nicely and leaving a small margin of surplus around the edges, it then should be removed and reinforced with plate or high karat solder to the desired thickness. After it is sufficiently reinforced to insure strength, replaced on tooth and riveted, the smooth marginal edges will enable it to be finished down to a fine line, leaving a joint, when finished, so close as to render it practically impervious; but in dressing it down along the cutting edge, the file should be passed on a parallel line with the face of the tooth, which will leave the thickness of the metal covering and protecting this edge, and which can be left of a uniform and desired thickness to receive the force of mastication and relieve the porcelain of all strain.

Another great annoyance, and of not uncommon occurrence, is the tendency of solder to ball up, and which is invariably caused by trying to make the solder to flow before the parts to be united are sufficiently heated. It is imperative to first gradually raise the heat of the higher fusing metal to or near the degree at which the solder will fuse. After accomplishing this, by applying the solder and then directing the flame on both the surfaces of the parts and of the solder having previously been fluxed with a thin paste of borax, no difficulty will be experienced in causing the solder to flow nicely with but a small pointed flame from the ordinary blowpipe, and without any exertion whatever. But if, on the contrary, the flame be directed on the solder before the parts to be united are sufficiently heated, it will fuse within itself and ball up, and if much time be thus consumed the baser alloy or zinc will be burned out, requiring, in consequence, a greater degree of heat to cause it to flow than otherwise, and as the depletion of the zinc will, to some extent, increase the fusing point and decrease its flowing properties, the liability of fusing or burning the object to be soldered is also increased.

The parts should be fluxed before heating up, and it is always necessary to flux well the surface of the solder before attempting to control it, as containing zinc renders it so easily oxidizable, it will not flow or cannot be managed unless it is properly fluxed. The borax should be mixed into a thin paste and applied with a thin camel's-hair brush, or similar means, as the quantity can be governed better and its application be made only where it is needed, thus avoiding the common and by far too generous use of powdered borax, which, as has been previously stated, increases the danger of cracking the teeth, renders the solder more difficult to handle, and oftentimes causes the surface of the work to be freely covered with small pits.

For investing there are perhaps several substances which, in conjunction with plaster, possess all the desired qualities and advantages; however, we are inclined to use and give preference to the use of a material composed of ordinary fine white lake sand, one-third, and plaster two-thirds, believing it to meet the requirements as well as any and much better than some, for being considerably coarser than many, it conducts the heat better, does not expand so readily, and the sand fusing so much higher than pumice-stone avoids the previously mentioned objectionable liability in connection with that substance.

The investment should be no larger in size or bulk than merely sufficient to cover and protect the teeth and retain the parts in position, and should be protected from expansion as much as possible by a narrow rim or ferrule of metal, or by being poured on to finely interlaced wire netting.

Where the parts to be soldered are not in contact one with the other, as frequently occurs, the spaces between may be filled in with gold foil to good advantage, and thus bridged across, render the work of uniting them very simple; or with the use of a pointed steel instrument, the solder, when just beginning to fuse, can be pulled or coaxed over and across such spaces with ease.

In coaxing and in the controlling and management of solder, it is of much material assistance to note that gravity be favorable, for however skilful one may become, it is difficult to cause it to run up hill against its own weight; consequently, in uniting a piece of two or more teeth for instance, and especially if it be for the anterior portion of of the mouth where the curvature is greater, it is necessary to change the relative position as you progress, that the solder may run where it is required to remain when fused.

Another not unusual occurrence of no little annoyance in the construction of a plate or crown, is the appearance of small holes in the metal during the process of annealing and swaging, and which is usually caused by a baser metal on the surface of the gold, usually tin or lead, which, fusing so much lower than the gold, becomes incorporated at its point of contact when being annealed. Treat the metal to the acid bath after each swaging, being careful afterwards to thoroughly neutralize the acid by the free application of water before again attempting to heat or solder.

When the burning of such a hole does occur, or when one presents itself during the process of finishing. it can easily be obliterated with the use of a solder of the same karat as the gold by sweating over it a piece little larger than the hole from the side not interfering with the fit or adaptation. It should not be the attempt to fuse the solder till it flows, but by first thoroughly cleaning and removing oxidation; investing if necessary to prevent unsoldering or for protection of porcelain; properly fluxing the surfaces of metal and solder; heating to a red heat and then simply wilting or sweating the solder down to its place by the use of a small pointed intermittent flame from the blowpipe only till it becomes firmly attached without flowing perceptibly, then ceasing; the desired result is accomplished with comparative ease and very little danger.

If no investment be used, or necessary, or even in cases where it is required, a precautionary means is frequently necessary to prevent the unsoldering of the parts; by covering or coating such surfaces with either a solution of whiting or of plumbago, carbon or crocus, any of which will relieve all danger from that source.

I am of the impression that so far as soldering is concerned, the use of the bellows is rarely if ever indicated, and should in fact be avoided; for the amount of heat necessary, which is usually very much overestimated by the way, cannot be so well regulated, and more harm than good is liable to result in consequence. A greater heat than the combination mouth governed blowpipe will give, if properly applied, is very seldom required, but to blow a continuous flame is a very valuable accomplishment for the dentist doing gold work, and can be learned by most any one devoting to it a little perseverance and application.—*Dental Review.*

